# The household economic burden of drug-susceptible TB diagnosis and treatment in The Gambia

**DOI:** 10.5588/ijtld.22.0091

**Published:** 2022-12-01

**Authors:** I. Devoid, A. K. Sillah, J. Sutherland, O. Owolabi, O. Ivanova, C. Govathson, K. Hirasen, M. Davies, K. Lönnroth, I. Loum, A. Touray, S. Charlambous, D. Evans, M. Quaife

**Affiliations:** 1 Faculty of Public Health and Policy, London School of Hygiene & Tropical Medicine, London, UK; 2 Medical Research Council Unit The Gambia at the London School of Hygiene & Tropical Medicine, Banjul, The Gambia; 3 Center for International Health, Ludwig-Maximilians-University (LMU) Munich University Hospital, Munich, Germany; 4 Division of Infectious Diseases and Tropical Medicine, Medical Centre of the University of Munich (LMU), Munich, Germany; 5 German Centre for Infection Research (DZIF), Partner Site Munich, Munich, Germany; 6 Health Economics and Epidemiology Research Office, Department of Internal Medicine, School of Clinical Medicine, University of the Witwatersrand, Johannesburg, South Africa; 7 Department of Public Health Sciences, Karolinska Institute, Sweden; 8 Aurum Institute, Johannesburg, South Africa

**Keywords:** tuberculosis, patient costs, catastrophic costs, The Gambia

## Abstract

**OBJECTIVE:**

To determine the costs and catastrophic costs incurred by drug-susceptible (DS) pulmonary TB patients in The Gambia.

**METHODS:**

This observational study collected cost and socio-economic data using a micro-costing approach from the household perspective from 244 adult DS-TB patients with pulmonary TB receiving treatment through the national treatment programme in The Gambia. We used data collected between 2017 and 2020 using an adapted version of the WHO generic patient cost survey instrument to estimate costs and the proportion of patients experiencing catastrophic costs (≥20% of household income).

**RESULTS:**

The mean total cost of the TB episode was $104.11 (2018 USD). Direct costs were highest before treatment ($22.93). Indirect costs accounted for over 50% of the entire episode costs. Using different income estimation approaches and catastrophic cost thresholds, 0.4–75% of participants encountered catastrophic costs, showing the variability of results given the different assumptions we utilised.

**CONCLUSIONS:**

We show that despite the benefits of free TB care and treatment, DS-TB patients still incur substantial direct and indirect costs, and cases of impoverishing expenditure varied vastly depending on the income estimation approaches used.

## Abstract (fr)

**OBJECTIF:**

Déterminer les coûts et les coûts catastrophiques assumés par les patients atteints de TB pulmonaire pharmacosensible (DS-TB) en Gambie.

**MÉTHODES:**

Cette étude observationnelle a recueilli les données de coûts et les données socio-économiques à l’aide d’une approche de micro-costing, en se concentrant sur le point de vue des foyers de 244 patients adultes atteints de DS-TB sous traitement via le programme national de traitement de Gambie. Nous avons utilisé les données recueillies de 2017 à 2020 en utilisant une version adaptée du Manuel d’enquête de l’OMS sur les coûts de la tuberculose pour les malades, afin d’estimer les coûts et la proportion de patients faisant face à des coûts catastrophiques (≥20% des revenus du foyer).

**RÉSULTATS:**

Le coût total moyen de l’épisode tuberculeux était de 104,11 $ (USD 2018). Les coûts directs étaient plus élevés avant traitement (22,93 $). Les coûts indirects représentaient plus de 50% de l’ensemble des coûts de l’épisode. En utilisant différentes approches d’estimation des revenus et des valeurs seuils pour les coûts catastrophiques, 0,4–75% des participants faisaient face à des coûts catastrophiques. Cela témoigne de la variabilité des résultats au vu des différentes méthodes d’estimations utilisées.

**CONCLUSIONS:**

Malgré les avantages d’une prise en charge et d’un traitement gratuits de la TB, les patients atteints de DS-TB font toujours face à des coûts indirects et directs conséquents, et les cas de dépenses appauvrissantes varient considérablement selon l’approche d’estimation des revenus utilisée.

Until the COVID-19 era, TB disease was the leading cause of death from a single infectious agent worldwide, and nearly half of patients living with TB in low- and middle-income countries (LMICs) reported financial difficulties due to the disease.^1^ TB patients encounter sizeable medical and non-medical costs from before diagnosis until after treatment, but also due to morbidity and disability that can lead to lost productivity during this time.^2^

The WHO End TB Strategy aims to eliminate TB-related catastrophic costs that patients and their families experience by 2030, and defines TB costs as “catastrophic” when they exceed 20% of annual pre-TB household income.^3^ Despite efforts from policy-makers and health systems to alleviate patient costs, many households still face catastrophic costs due to TB.^1^,^4^ TB treatment is often provided free at the point of access in many settings. While there is information regarding catastrophic costs for high TB burden countries, there is limited information for The Gambia, which has a relatively low incidence of TB, but ranks among the poorest in the world with a gross national income per capita of less than $1,500.^5^

The majority of patients initiate care at government (public) health facilities in The Gambia, incur no charges for diagnostic GeneXpert testing and are then treated at governmental facilities, where they incur no cost. However, patients who require additional tests such as X-rays or other imaging must pay out-of-pocket expenses at private pharmacies, clinics or hospitals. Patients also incur non-medical costs through expenditure on transport, food, accommodation and indirect costs due to an inability to work or perform daily activities.^6^

Substantial patient costs can impede those most vulnerable from accessing essential care and treatment services due to an inability to pay, and ultimately, exacerbate TB transmission.^3^ Information on costs and the proportion facing catastrophic costs are vital to inform social protection programmes to ensure that patients receive appropriate care and treatment, and are supported without the risk of financial catastrophe.

The WHO acknowledges several methods of estimating household capacity to pay.^3^ A study from Sweeney et al. showed that catastrophic cost estimates varied vastly using different income estimation approaches, which displayed the diverse results and policy implications that can occur from use of distinct methodologies from the TB patient costing literature.^7^

This study has both empirical and methodological contributions. First, while many studies have estimated the proportion of those who experience catastrophic costs due to TB in LMICs,^1^,^4^, there is a lack of information regarding patient costs and catastrophic costs suffered due to the use of TB care in The Gambia.^4^ Second, few studies explore the impact of different assumptions of household capacity to pay on catastrophic cost estimates. In the present study, we estimate the direct, indirect and catastrophic costs incurred by DS-TB patients in The Gambia, exploring the impact of different methodological assumptions on these estimates.

## METHODS

### Setting, sites, and study participants

Data were collected at the Medical Research Council Unit The Gambia of the London School of Hygiene & Tropical Medicine (MRCG@LSHTM) in the Greater Banjul Area (GBA), where nearly two-thirds of TB patients in The Gambia are diagnosed.^8^ The GBA is a mixed urban and rural setting of approximately 700,000 inhabitants.^8^ In 2019, the incidence rate of TB in The Gambia was 174 cases per 100,000 population.^9^

Ethics approval was obtained from The Gambia Government/MRC Joint Ethics Committee on 17 January 2018 (Ethical approval number for TB Sequel: SCC1523).

This study used longitudinal data that were collected as part of the TB Sequel study (NCT03251196).^10^ One of this study’s aims is to describe the economic costs of pulmonary TB to patients and their households. Adults (≥18 years) seeking care through the national TB programme in The Gambia who provided written informed consent, living within the study area, willing to be tested for HIV, with available collected and stored bodily fluids, and willing to start anti-TB treatment after diagnosis were eligible to participate. Patients were recruited from government health facilities or referred to the MRCG@LSHTM. Eligible participants were enrolled at TB treatment initiation between 2017 and 2020. Direct (medical and non-medical) and indirect costs data were collected using the WHO generic patient cost instrument adapted for use in a longitudinal study.^11^ The surveys were administered by trained members of the TB Sequel study. Patients were surveyed at enrolment into the study and at the end of the intensive and continuation phases of treatment. The surveys comprised of questions on socio-economic and household information such as healthcare-seeking behaviours, costs of care, income, asset ownership and financial coping mechanisms.^10^ Responses were entered on paper forms and later captured into an online data collection tool in OpenClinica® (https://www.openclinica.com/). We restricted the analysis to patients enrolled with DS-TB and used a micro-costing approach from the household perspective to estimate patient costs incurred before treatment starts and while on TB treatment. Micro-costing is a method to capture all cost components of healthcare services or interventions more precisely.^12^ Participants who did not attend at all three visits or tested positive for rifampicin-resistant TB were excluded from the analysis.

### Data analysis

#### Costs incurred by drug-susceptible TB patients

Total direct costs were summed and disaggregated by costing category and treatment phase. Direct costs were categorised as medical (consultation, imaging, medication, hospitalisation), non-medical (transport, food [including the cost of nutritional supplements], accommodation), associated with seeking TB care.^3^ At enrolment, patients recalled the total costs incurred before treatment at each provider by cost category. In the pre-treatment phase, we assessed the frequency, duration and costs incurred by different provider types. In the intensive and continuation treatment stages, the same costing categories were used for TB-related follow-up visits, hospitalisation or DOTS visits.

Indirect costs were calculated using the human-capital approach, where the total time participants spent seeking care and treatment for TB was estimated and classified as travel, waiting, consultation and hospitalised time or guardian costs (costs or time incurred by family). The average number of visits per participant was multiplied by the mean visit length per participant within the respective treatment phase. An hourly wage was estimated by dividing monthly individual income by reported monthly working hours, and the total time lost was multiplied by the hourly wage per participant to calculate the total indirect cost (WHO method).^3^

Direct costs and income reported before 2018 were inflated to 2018, using the 2018 World Bank GDP deflator, and converted to USD using the conversion rate in January 2018 (Gambian dalasi [GMD] 47.31 = 1USD).^13^,^14^ Costs incurred after 2018 were not inflated. Analyses were performed using Stata v16.1 (StataCorp, College Station, TX, USA) and MS Excel (MicroSoft, Redmond, WA, USA).^15^,^16^

### Missing cost or income data

Missing cost data were replaced with zero for patients who attended the survey visit, assuming no cost was incurred,^17^ as the study team noted that when patients incurred no cost at a provider or category, the interviewers left it blank instead of denoting a value of zero ( Supplementary Data).

### Calculation of catastrophic costs

Catastrophic costs were estimated by summing total direct and indirect costs throughout the TB episode divided by annual household income (Pre-TB). Predicted household income based on asset ownership and housing characteristics was used to serve as the default approach of catastrophic cost estimation. Participants were asked about asset ownership, and the same asset and housing characteristics as the 2010 World Bank Integrated Household Survey (IHS) were used to perform a principal components analysis (PCA) to calculate factor weights for each asset or housing characteristic in the IHS,^18^ including washing machine, landline phone, cellphone, car, motorcycle, bicycle, refrigerator, television, DVD player, radio, horse/donkey, computer and housing characteristics, which included water source, electricity and toilet type. The factor weights from the first dimension of the PCA in the 2010 IHS dataset were applied to the study dataset to construct nationally representative wealth quintiles, to explore the socio-economic status of the patients in this dataset. The mean permanent household income from each quintile of the IHS was computed and applied to the corresponding quintile within the TB Sequel data to predict household income for each participant (Approach #1).^7^,^19^ A catastrophic cost threshold of 20% was used, and varied between 5% and 30%.^3^

### Sensitivity analyses

The indirect costing methods were assessed for variation in results using an approach from Sweeney et al., according to which monthly household income (for each of the three different income estimation approaches we use) was divided by the total number of reported adults in the household, which was divided by self-reported monthly working hours to estimate the hourly wage. The estimated hourly wage was then multiplied by the total lost time to calculate the total indirect cost.^7^

To enumerate household income, we assumed each household earns the national mean monthly income from the 2010 IHS. Second, we utilised a method in the patient costing literature where the catastrophic costs threshold was defined when total costs exceeded 10% of individual annual income, and income was imputed as $1 when zero or missing.^20^–^23^ We also calculated the proportion of patients who used coping methods, including taking loans, selling assets or borrowing money to cover out-of-pocket costs related to TB care, which assumes any household using a coping mechanism has experienced catastrophic cost in line with the 2019 WHO Handbook.^3^ Finally, sensitivity analyses were performed by adjusting the catastrophic cost threshold between 5% and 30%.^7^

## RESULTS

### Baseline characteristics

Table 1 displays the characteristics of the 244 DS-TB patients who were included for analysis in the study (Supplementary Data). The mean age of the included participants was 33 years; 71% of participants were adult men, roughly in line with the proportion of TB cases in The Gambia (57%).^9^ The proportion of HIVTB co-infected participants (8%) was lower than the HIV-TB co-infection rate in The Gambia (16%).^24^ The reported unemployment rate for study participants (34%) was approximately three-fold the national unemployment rate in The Gambia (9%).^25^ Respectively 11% and 83% of participants had either lost their jobs or were working less at study enrolment. Based on results from the PCA, respectively 45% and 30% of included study participants fell into the richest/wealthiest quintiles 4 and 5.

**Table 1:** Participant characteristics (n = 244).

Factor	Included individuals (*n* = 244) *n* (%)
Age group, years	
18–29	115 (48)
30–40	67 (28)
≥41	59 (24)
Sex	
Female	71 (29)
Male	173 (71)
Education	
No formal schooling	83 (34)
Primary school	34 (14)
Secondary school	59 (24)
High school	68 (28)
HIV status	
HIV-positive	20 (8)
HIV-negative	221 (92)
Employment at enrolment	
Self-employed	38 (16)
Formally employed	22 (9)
Informally employed	28 (11)
Unemployed	83 (34)
On sick leave	51 (21)
Student	21 (9)
Household primary income earner at enrolment	
Patient	79 (33)
Immediate family member	63 (26)
Extended family member	36 (15)
Other	64 (26)
Job loss at study enrolment	
No	218 (89)
Yes, lost job	26 (11)
Working less than normal at enrolment	
No	41 (17)
Yes, less work	203 (83)
Socio-economic status	
1 – poorest	18 (7)
2	32 (13)
3	11 (5)
4	109 (45)
5 – richest	74 (30)
Number of weeks from symptom onset to seeking care, mean ± SD	6.2 ± 5.12

SD = standard deviation.

### Direct and indirect costs

**Figure 1:**
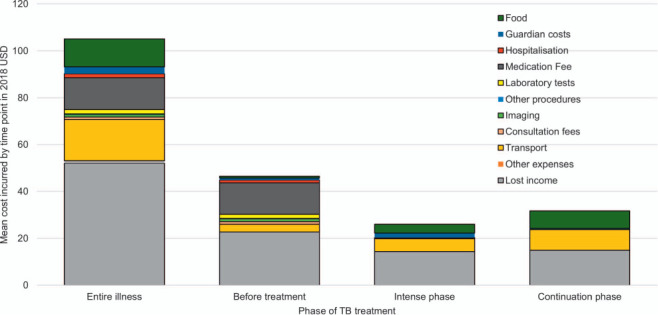
Mean direct and indirect cost per category and time-period in 2018 USD (n = 244). Lost income calculated using the permanent income estimation approach and the WHO method of hourly wage valuation. The intensive treatment phase was 8 weeks, and the continuation phase was 16 weeks. USD = US dollar.

Overall, mean direct costs before treatment accounted for the highest proportion of direct costs throughout the TB episode (47%). 59% ($13.49) of the mean direct costs before treatment comprised of medication fees. In the intensive phase, the mean direct cost was $9.77, and patients incurred little to no direct medical costs due to existing TB treatment coverage.^6^ Furthermore, the mean direct cost in the continuation phase was $16.30. Figure 1 exhibits the mean direct and indirect costs for the entire TB episode. The mean total cost for the TB episode was $104.11. The mean direct cost for the entire TB episode was $49.00, and the costliest component was transport ($17.62).

**Figure 2:**
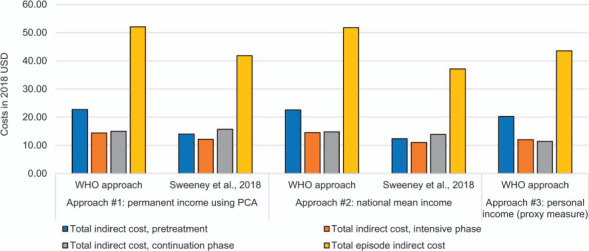
Indirect cost by time-period and income estimation approach in 2018 USD. Approach #1: income calculated using a principal components analysis; Approach #2: income calculated using the national mean income of The Gambia; Approach #3: income calculated using self-reported personal income. Where missing income was imputed as $1 (2018 USD). The WHO Approach was calculated using the human-capital approach. Sweeney et al. divided monthly household income by the total number of reported adults in the household, which was divided by self-reported monthly working hours to estimate the hourly wage. USD = US dollar; PCA = principal components analysis.

Using the WHO approach of hourly wage valuation and permanent income, the mean indirect cost throughout the TB episode was $52.06. Figure 2 displays the average indirect costs incurred by time point and use of sensitivity analyses surrounding income and methodology of hourly wage valuation. Total mean indirect costs were lowest using mean national income and the approach from Sweeney et al. ($37.14).^7^ Table 2 gives the total mean and median direct costs, provider visits and total time spent seeking care by provider type before treatment. The most substantial mean direct costs and total time lost was incurred at the traditional practitioners where patients spent $30.80 and exhausted 50 h seeking care on average, followed by private practitioners where patients sustained $14.84 and expended 5 h seeking care on average.

**Table 2:** Mean visit number, direct costs and hours seeking care before treatment by provider type in 2018 USD (n = 244)

Facility type*	Mean total number visits *n*	Median total time spent seeking care (hours)	Total direct costs per provider (in 2018 USD)	

			Mean ± SD	Median [IQR]
Pharmacy, drug or grocery store (*n* = 115)	2	3	11.48 ± 14.28	6.87 [3.70–12.89]
Traditional practitioner (*n* = 32)	2	50	30.80 ± 40.70	12.84 [4.17–43.45]
Primary care, public (*n* = 78)	2	5	4.46 ± 7.85	2.09 [0.87–5.39]
Private practitioner (*n* = 39)	2	5	14.84 ± 28.66	6.13 [1.94–12.39]
Public hospital (*n* = 191)	2	8	5.13 ± 6.73	2.56 [0.95–7.21]
Private/mission hospital (*n* = 214)	1	11	7.52 ± 26.41	2.86 [0–5.39]
Other (*n* = 2)	2	3	4.49 ± 6.35	4.49 [0–8.98]

* Total number of included participants who attended visits at the specified provider.

USD = US dollar; SD = standard deviation; IQR = interquartile range.

### Catastrophic costs

**Figure 3:**
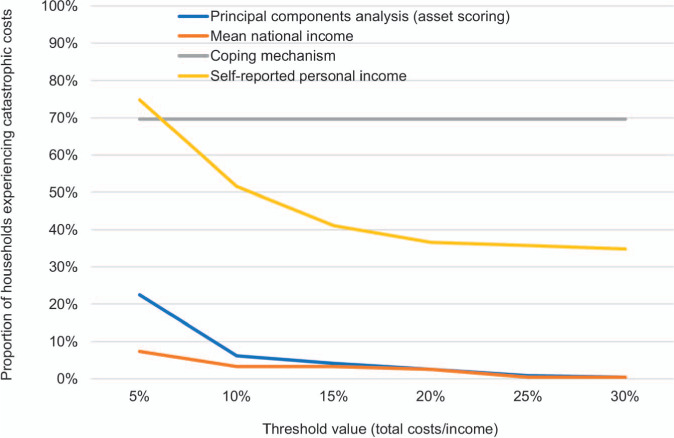
Catastrophic cost prevalence by threshold value and income variation approach (n = 244). Indirect costs calculated using the human capital approach and the WHO method of hourly wage valuation. Approach #1: income calculated using permanent income; Approach #2: income calculated using mean national income; Approach #3: income calculated using self-reported personal income (missing income was imputed as $1 USD). PCA = principal components analysis; USD = US dollar.

Figure 3 illustrates the proportion of catastrophic costs experienced using different income estimation approaches and catastrophic cost thresholds. Participants experienced catastrophic costs ranging from 0.4% to 75%, using the aforementioned approaches; this highlights the substantial variability in catastrophic cost estimates depending on the assumptions used. At the 20% standard catastrophic cost threshold, approximately 3% of participants experienced catastrophic costs using both permanent income and mean national income. Participants experienced the highest proportion of catastrophic costs when using self-reported individual income (35–75%).

**Figure 4:**
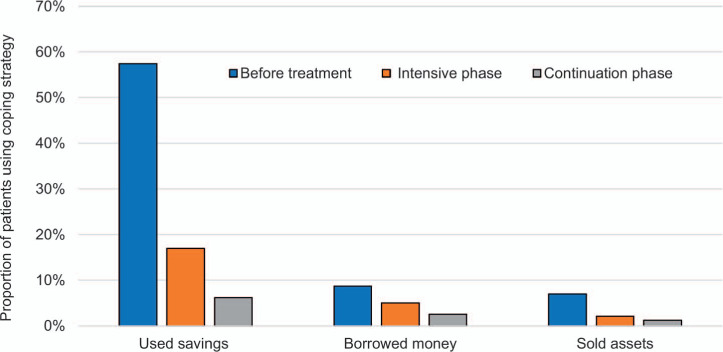
Proportion of patients employing different coping strategies by time point (n = 244).

Figure 4 displays the proportion of patients utilising coping strategies by time-period and strategy type. Nearly 70% of participants used any method of dissaving during the episode, and were most prevalent before treatment, where participants incurred the highest direct costs.

## DISCUSSION

Despite treatment being free at the point of access, this study suggests DS-TB patients can incur substantial and possibly catastrophic costs during a TB episode in The Gambia. The mean cost of the TB episode to the patient exceeded $100 for the 244 patients included in this analysis. Before treatment, patients incurred the highest direct costs by treatment stage; these accounted for nearly 47% of total direct expenses, and 22% of total episode costs. These findings are similar to those of a systematic review on TB patient costing studies across LMICs, which found total episode costs to range from $55 to $8,198, demonstrating the wide range of patient costs experienced across LMICs.^1^ The mean total costs from this study are on the lower end of this range; however, the breakdown of results are in line with other TB patient costing studies in LMICs and sub-Saharan Africa, where patients incurred the highest proportion of direct costs before treatment, and few participants experienced direct medical expenses during the intensive or continuation phases of treatment due to existing treatment policies.^1^,^20^,^26^–^28^ Overall, the high direct-medical costs before treatment and direct non-medical costs during treatment stages mirrors the actual trends in costs faced by TB patients in sub-Saharan Africa and LMICs.^1^,^4^

Depending on the approach used to estimate income, the proportion of patients experiencing catastrophic costs varied greatly. Using permanent or mean national income, the catastrophic costs estimates resemble those in Sweeney et al.,^7^ where few participants experienced catastrophic costs, but a much higher proportion of participants reported the use of coping strategies. The catastrophic cost estimates using individual income and a 10% threshold are comparable to those in Foster et al.,^20^ which also used individual income and a 10% threshold, where 53% of TB participants experienced catastrophic costs compared to 52% of participants in this study. The proportion of patients using coping strategies in this study was much higher than in Sweeney et al.,^7^ Pedrazzoli et al.,^29^ and Foster et al.,^20^ plausibly due the high unemployment rate, difference in study setting or limited recall bias from longitudinal surveys. A recent study in Uganda,^30^ where the GDP per capita and TB incidence are similar to The Gambia, found a higher range of costs and catastrophic costs than our study. However, a study from Malawi, where the GDP per capita and TB incidence are also similar,^31^ reported total patient costs that were comparable to those in our study.

Nonetheless, cross-country catastrophic cost comparisons are difficult to make due to the variety of approaches utilised in the literature to enumerate household income and patient costs. Such approaches and the assumptions used to obtain estimates can vastly differ; this highlights the importance of sensitivity analyses surrounding household ability to pay. This study reinforces the findings of Sweeney et al.^7^—that the methods used to estimate income significantly affect catastrophic cost estimates.

The use of consumption or expenditure is the most accurate method of enumerating household capacity to pay and would increase the validity surrounding the catastrophic cost estimates presented in this study. However, consumption and expenditure surveys are time-consuming and still subject to recall and social desirability bias.^22^,^32^ In lieu of data on consumption or expenditure, it is challenging to obtain precise estimates of catastrophic costs using measures such as self-reported household income, permanent income and other proxy measures. Future TB patient costing studies could utilise consumption or expenditure to measure household income; however, the time taken to conduct such surveys could lead to misclassification of other survey elements like cost and socioeconomic information due to an increased level of survey fatigue.^7^

Our use of longitudinal surveys is plausibly less prone to recall bias, whereas most patient costing studies use one survey conducted at the end of treatment. This was especially true before treatment, where our study was able to capture a variety of indicators on patients’ care-seeking behaviours.

This study had several limitations. For enrolment in our study, patients needed to be diagnosed and have started treatment; thus, we potentially excluded those who died or were lost to follow-up before treatment could be started. Such patients are perhaps most prone to financial catastrophe, which could have led to an underestimation of the catastrophic cost prevalence. Therefore, this study gives a true estimation of patient and catastrophic costs for those who were able to engage in care for the entire TB episode, as the same barriers limiting those in the richer/wealthier quintiles from accessing care, could likely have limited those in the poorest quintile from ever accessing care.

Indirect costs were only estimated using the human capital approach, which can undervalue groups like the unemployed, informal workers and ageing populations.^33^ The cohort had a large percentage of unemployed and informal workers, which could have led to an underestimation of indirect costs, as income data are likely not missing at random and are subject to social desirability and recall bias. Moreover, missing direct cost data were imputed with the value zero on the assumption that participants had not incurred any direct cost during this period, which may have resulted in an underestimation of direct costs of the TB episode, and thereby, an underestimation of the proportion of participants incurring catastrophic costs. The MRCG@LSHTM stopped study activities on 27 March 2020 due to COVID-19, and in-person interviews were not resumed until September 2020.

The high costs experienced before treatment demonstrate the need for policy shifts to relieve the burden patients experience pre-TB diagnosis. Community education on TB symptoms along with adequate access to affordable diagnostics could potentially alleviate the 6-week period from diagnosis to treatment. Such approaches could ensure patients are diagnosed early, thus, saving money on non-governmental facility visits, where large expenditures often occur. Integrating such facilities into the continuum of care could shorten the period from symptom onset to diagnosis. Two systematic reviews found better outcomes and linkage to care through public-private partnerships,^34^,^35^ but further research is needed to fully understand how to integrate traditional practicioners where patients exhausted sizeable direct and indirect costs.

## CONCLUSION

This is the first study to assess the economic burden of TB in The Gambia. Patients can experience large costs associated with DS-TB, despite free treatment in The Gambia. Catastrophic costs varied vastly according to the approach taken to estimate income; this stresses the importance for future research of using a variety of approaches and acknowledging biases from such approaches carefully.
