# Supplementary material for: The household economic burden of drug-susceptible TB diagnosis and treatment in The Gambia
Source: Int J Tuberc Lung Dis. 2022 Dec 1;26(12):1162–9. doi: 10.5588/ijtld.22.0091 (PMC9728947; doi:10.5588/ijtld.22.0091)
Supplement: Supplementary file 1 [file iutld_ijtld_22.0091_supplementarydata1.pdf]

http://dx.doi.org/10.5588/ijtld.22.0091

SUPPLEMENTARY DATA

The household economic burden of drug-susceptible TB diagnosis and treatment in The Gambia

Missing cost or income data:

Missing individual income was assumed missing at random and imputed with the mean hourly wage for participants who it was available if missing or zero. When participants could not report the individual costing categories, the proportional breakdown of costs from the respective treatment phase was extrapolated across the costing categories for patients whom full breakdowns were available per WHO guidelines (3).

Baseline Characteristics:

139 participants were initially dropped due having no baseline data available (n=49), testing negative for TB (n=10), testing positive for multi-drug resistant TB (RR-TB) (n=3) or inability to attend any of the baseline or follow-up visits (n=77), leaving 244 DS-TB patients included for analysis in the study.

Surveys:

Surveys used for data collection available at: https://www.tbsequel.org/resources/materials/

Total mean and median direct costs by time-period and cost category in 2018 $USD (N=244)

	Entire Illness		Before Treatment		Intensive Phase		Continuation Phase	
**Item**	**Mean (Std. dev)**	**Median *(IQR)**	**Mean (Std. dev)**	**Median (IQR)**	**Mean (Std. dev)**	**Median (IQR)**	**Mean (Std. dev)**	**Median (IQR)**
**Other expenses**	0.02 (0.33)	-	0.02 (0.33)	-	-	-	-	-
**Accommodation**	-	-	-	-	-	-	-	-
**Food**	11.97 (20.66)	3.38 (0 - 14.27)	0.77 (1.93)	0 (0 - 0.74)	3.79 (8.84)	0 (0 - 1.90)	7.41 (14.80)	0 (0 - 8.45)
**Transport**	17.62 (16.61)	13.05 (7.58 - 24.36)	3.32 (6.34)	1.52 (0.68 - 3.21)	5.50 (6.93)	2.71 (0 - 9.17)	8.80 (12.45)	5.41 (0 - 10.82)
**Consultation Fee**	1.08 (2.68)	0.53 (0 - 1.06)	1.07 (2.68)	0.53 (0 - 1.06)	0.01 (0.03)	-	-	-
**Imaging**	1.13 (2.10)	0 (0 - 2.11)	1.13 (2.10)	0 (0 - 2.11)	-	-	-	-
**Other procedures**	0.19 (1.72)	-	0.19 (1.72)	-	-	-	-	-
**Lab tests**	1.85 (4.54)	0 (0 - 1.27)	1.75 (4.37)	0 (0 - 1.06)	0.01 (.08)	-	0.087 (1.36)	-
**Medication Fee**	13.49 (23.87)	5.39 (0.53-13.21)	13.48 (23.87)	5.39 (0.53 - 13.21)	0.01 (.068)	-	0.001 (0.01)	-
**Hospitalization**	1.65 (17.48)	-	1.20 (16.15)	-	0.45 (6.82)	-	-	-
**Guardian Costs**	3.05 (11.90)	0.34 (0 - 1.90)	0.89 (1.76)	0 (0-1.14)	1.82 (11.67)	-	0.34 (1.63)	-
****Lost Income**	52.06 (154.32)	20.24 (10.09 – 40.29)	22.69 (136.31)	6.72 (2.59 – 11.77	14.40 (46.55)	4.25 (1.59 - 11.70)	14.97 (35.96)	6.17 (1.86 - 13.15)
**Total Episode Cost**	104.11 (175.72)	62.51 (38.02 – 111.60)	46.51 (155.91)	19.88 (11.43 – 38.26)	25.99 (52.41)	9.97 (4.80 – 26.67)	31.61 (44.91)	17.23 (7.38 – 37.60)

**IQR: Interquartile range*

***Calculated using WHO approach of hourly wage valuation and permanent income*
